# PROspective MEmory Training to improve HEart failUre Self-care (PROMETHEUS): study protocol for a randomised controlled trial

**DOI:** 10.1186/s13063-015-0721-2

**Published:** 2015-04-29

**Authors:** Jan Cameron, Peter G Rendell, Chantal F Ski, Christina E Kure, Skye N McLennan, Nathan S Rose, David L Prior, David R Thompson

**Affiliations:** Centre for the Heart and Mind, Mary MacKillop Institute for Health Research, Australian Catholic University, Level 5, 215 Spring Street, Melbourne, VIC 3000 Australia; Cognition and Emotion Research Centre, School of Psychology, Australian Catholic University, 115 Victoria Parade, Melbourne, VIC 3065 Australia; Department of Psychiatry, University of Wisconsin, 6001 Research Park Boulevard, Madison, WI 53179 USA; Department of Cardiology, St Vincent’s Hospital, Princess Street, Melbourne, VIC 3065 Australia

**Keywords:** Chronic heart failure, Self-care, Cognitive function, Prospective memory, Cognitive training, Randomised controlled trial

## Abstract

**Background:**

Cognitive impairment is seen in up to three quarters of heart failure (HF) patients and has a significant negative impact on patients’ health outcomes. Prospective memory, which is defined as memory to carry out future intentions, is important for functional independence in older adults and involves application of multiple cognitive processes that are often impaired in HF patients. The objective of this study is to examine the effects of prospective memory training on patients’ engagement in HF self-care and health outcomes, carer strain and quality of life.

**Methods/design:**

The proposed study is a randomised, controlled trial in which 200 patients diagnosed with HF, and their carers will be recruited from 3 major hospitals across Melbourne. Eligible patients with HF will be randomised to receive either: 1) The Virtual Week Training Program - a computerised prospective memory (PM) training program (intervention) or 2) non-adaptive computer-based word puzzles (active control). HF patients’ baseline cognitive function will be compared to a healthy control group (n = 60) living independently in the community. Patients will undergo a comprehensive assessment of PM, neuropsychological functioning, self-care, physical, and emotional functioning. Assessments will take place at baseline, 4 weeks and 12 months following intervention. Carers will complete measures assessing quality of life, strain, perceived control in the management of the patients’ HF symptoms, and ratings of the patients’ level of engagement in HF self-care behaviours.

**Discussion:**

If the Virtual Week Training Program is effective in improving: 1) prospective memory; 2) self-care behaviours, and 3) wellbeing in HF patients, this study will enhance our understanding of impaired cognitive processes in HF and potentially is a mechanism to reduce healthcare costs.

**Trial registration:**

Australian New Zealand Clinical Trials Registry #366376; 27 May 2014. https://www.anzctr.org.au/Trial/Registration/TrialReview.aspx?id=366376&isClinicalTrial=False.

## Background

Heart failure (HF) is a chronic syndrome with multiple symptoms that impose a significant burden on the individual and healthcare system [1]. The prevalence of HF is estimated at 23 million individuals worldwide and despite advances in management there remains substantial risk for high morbidity and mortality [2]. Cognitive impairment, which has been found to occur in up to 75% of HF patients [3], is a comorbidity that is gaining increased recognition as adding to HF morbidity and mortality [4,5].

There is compelling evidence demonstrating that HF patients are more vulnerable to changes in brain processes (memory, thinking, and thoughtful decisions) than similar matched individuals without HF [6]. Furthermore, almost three quarters of HF patients have evidence of cognitive impairments, especially memory, when brief cognitive screening questionnaires have been applied [3]. The impact of cognitive impairment in this vulnerable group of patients is significant as it also contributes to worse health outcomes, poor engagement in self-care and increased mortality [5,7,8].

Optimal self-care is considered an important non-pharmacological aspect of HF management that stabilises symptoms and improves health outcomes [7]. Key self-care behaviours include adhering to prescribed HF therapies including medications, dietary sodium and fluid restrictions, and exercise [9]. As well as adhering to these HF therapies, patients are taught to recognise and manage changes in symptoms, and seek health advice when such changes occur [9].

The significant research attention addressing methods to optimise HF self-care has also drawn attention to a host of barriers that have the potential to impede achievement of this outcome [10,11]. In recognising these barriers, it may not be feasible for all patients to develop adequate self-care skills [12].

Cognitive impairment is a key barrier to HF patients’ self-care as it has a negative impact on patient’s ability to engage in effective self-care behaviours, including initiating an appropriate and timely response to changes in HF symptoms [5,13]. The patient’s cognitive ability to respond to vital cues and initiate appropriate actions is not only critical in predicting engagement in self-care, it is frequently overlooked, with poor engagement in HF self-care often considered to reflect poor motivation and/or compliance [5].

Particularly relevant to cognitive ability is prospective memory (PM), currently a topic of intense interest in ageing and neuropsychology [14,15], although as yet given limited attention from the standpoint of HF. Described as remembering to carry out future intentions [16], PM includes many of the cognitive functions necessary in forming and initiating HF self-care decisions. One example specific to HF patients is that they: i) form the intention to weigh themselves daily, ii) hold on to this intention and conduct this behaviour each morning, and iii) initiate the appropriate action (reduce daily intake of sodium and fluids) in the event of a > 2-kg weight change. Thus, PM is not so much one specific cognitive process, but a set of cognitive processes involved in forming, retaining, initiating and executing an intention [17]. Successfully performing PM tasks requires intact functioning of multiple cognitive domains that are often impaired in HF patients [8]. These domains primarily include: attention; working memory; retrospective memory; and executive functioning [18].

The three leading theories as to why HF patients are vulnerable to cognitive changes are: i) chronic or intermittent hypoperfusion leading to cerebral ischaemia [19]; ii) microemboli causing cerebral infarction [20]; and iii) hypoxia arising from sleep apnoea [21,22]. However, in a recent review on cognitive impairment and cardiovascular disease [23] it was acknowledged that normal brain function requires seven interlinking physiological aspects to be intact: heart; lungs; elastic vessels; baroreflex; cerebrovascular arteries; small cerebral vessels; and cerebrospinal venous system. Conditions affecting these cardiovascular organs are often mutually interrelated, such that when one is anatomically or functionally altered it can lead to impaired cerebrovascular perfusion and increased risk of neurocognitive impairment. In functional neuroimaging studies of HF patients the most frequently damaged areas are the cortical white matter, particularly in the frontal lobes, and subcortical grey matter nuclei, particularly the thalamus, basal ganglia and brainstem [24-27]. The neural substrates in these regions of the brain are responsible for cognitive control operations and executive control. However, the temporal lobes of the brain may also be disrupted [26]. The temporal lobes play a key role in retrospective memory, which is critical for PM. Based on current evidence, it is likely that HF patients will have significant deficits in PM that are likely to impact on their everyday functioning.

Studies have demonstrated that PM is critical for maintaining functional independence in older adults [28] and PM tasks are sensitive to abnormal ageing [29]. The PM literature distinguishes between event-based tasks (for example, taking extra diuretic if weight changes by > 2 kg) and time-based tasks (for example, weighing at set times). One of the most valid assessment tools for measuring PM is Virtual Week; a computer program designed to simulate real world activities to test PM [30]. In preliminary analysis using Virtual Week to assess PM, we found that, compared with aged-matched healthy controls, HF patients had significant deficits across all types of PM tasks, even on those that minimised the demands for remembering the content of the task [31]. Because of the potential impact on health outcomes, PM represents a promising domain for cognitive training [32]. While much of the PM research has focused on ageing [28,29], and even though it is established that cognitive ability is often limited in HF patients, research to date is yet to investigate PM among this clinical cohort. This study is the first of its kind to do so and, in addition, to identify the potential of cognitive training to promote HF self-care. Further, we aim to examine whether carer burden (strain, perceived control and quality of life) changes in line with changes in PM of the HF patient.

In the area of cognitive training two main approaches have typically been investigated: 1) strategy-based training, which aims to compensate for limitations in underlying cognitive processes, and 2) process-based training, which aims to restore underlying cognitive processes that are impaired. These training studies have improved performance on the specific tasks used in training (near transfer) but have resulted in limited evidence for training gains to transfer to activities that are not closely related to the training task (far transfer) [33]. Furthermore, recent reviews have argued that cognitive training programs have typically implemented tasks that bear little resemblance to everyday life. For example, a working memory task such as learning a list of words may have little resemblance to adhering to HF self-care behaviours such as daily weighing and taking prescribed medications. Researchers have advocated the need for more ecologically valid training programs that target abilities such as PM [32], which is critical for maintaining functional independence [28]. Despite the importance of PM in daily life, there have been only a few small-scale training studies that have targeted PM [33]. The Virtual Week Training Program is a computerised program designed to simulate real world activities to test and improve PM [30]. The Virtual Week Training Program is a computerised extension of the standard Virtual Week program for assessing PM [30] that has a board game format with each circuit of the board simulating a day. Embedded within the game are hypothetical PM tasks that closely represent PM tasks in daily life, such as taking medication with meals and attending appointments at specific times (for example, 5 pm).

Preliminary findings using a 4-week version of the Virtual Week Training Program with healthy older adults had promising effects for both laboratory-based PM skills practiced (near transfer) and pragmatic PM tasks performed in everyday life (far transfer) [33]. Fifty healthy older adults participated in either the Virtual Week Training Program, an active control (involving music training) or a passive control. After the training, participants in the Virtual Week training group showed greater improvement in a real-life PM task, the call-back task, compared to both no-contact and active control groups, suggesting there was some transfer to performing PM tasks in the real world. Furthermore, participants in the Virtual Week training group showed a greater pre- to post-test improvement in Timed Instrumental Activities of Daily Living (TIADL) after PM training compared to both control groups, indicating far transfer [33]. We aim to extend these preliminary findings by examining the efficacy of a 6-week PM training program using the Virtual Week Training Program among HF patients. The impact of Virtual Week training will also be examined with regard to carer strain and quality of life. Furthermore, our comprehensive assessments of cognitive functioning will enable us to examine near to far training benefits in everyday functioning.

### Rationale for study

Although cognitive impairment among HF patients has received considerable attention, this will be the first study to specifically examine PM among this clinical group. This study will add evidence for the impact of cognitive impairment on HF patients and their carers by examining how PM relates to patient engagement in HF self-care and health outcomes. This study will also provide evidence for the feasibility of a novel computer-based memory training program (the Virtual Week Training Program) and will test whether this improves functional outcomes among HF patients.

### Study aims and objectives

We will conduct a randomised controlled trial to:

1. compare and confirm cognitive deficits, including PM deficits, of HF patients relative to healthy matched control group;
2. examine if PM is a significant predictor of HF self-care;
3. test the feasibility of implementing the innovative restorative memory training;
4. examine if PM training, using the Virtual Week Training Program, improves self-care and functional outcomes among HF patients (near to far transfer effects) and reduces hospital readmissions;
5. examine if PM training of HF patients changes their careers’ burden (strain, perceived control and contribution to patient HF self-care) and enhances their quality of life.

### Hypotheses

We hypothesise that the cognitive training will result in improved functioning, better health outcomes and reduced healthcare costs in this patient group. In addition, we predict that the improved functioning of the HF patients will reduce levels of carer burden (strain, perceived control, contribution to patient HF self-care) and improve their quality of life.

## Methods/design

### Study design

This will be a randomised controlled trial where 200 patients with HF will be randomised to receive either: 1) the Virtual Week Training Program, a computerised adaptively difficult PM training program, or 2) non-adaptive computer-based word puzzles. Sixty healthy control participants, with no HF diagnosis, will also be assessed to compare differences in baseline measures of cognitive and psychosocial functioning. There are 4 phases to the study: 1) baseline testing; 2) 6 weeks of cognitive training; 3) Assessment One, 2 to 4 weeks post cognitive training; and 4) Assessment Two, 12 months post cognitive training. There will be two separate interviews during each of the assessments (baseline testing, Assessment One and Assessment Two). These will be: a) nursing assessment including physical functioning, and self-care; and b) PM and neurocognitive assessment, and TIADL.

### Setting

Participants will be recruited from three major metropolitan hospitals in Melbourne, Australia.

### Participant inclusion criteria

A total of 200 HF patients aged 18 years or older will be recruited. Inclusion criteria will be: diagnosis of HF with reduced ejection fraction (ejection fraction < 45% in the previous 2 years) as confirmed by a cardiologist and consistent with Australian guidelines [34]; attendance at a specialist HF clinic or rehabilitation program, and/or a disease management program with nursing follow-up and able to participate in the study (that is adequate vision, hearing and English comprehension). Carers will be identified by HF patients as someone who is familiar with and contributes to the management of their heart condition (for example, a partner, family member, friend or next of kin). Patients who cannot identify a carer will still be eligible to participate in the study. All HF patients enrolled in the study will continue to receive usual care which can be from any combination of nurse-led chronic disease management programs, specialised HF outpatient clinics, or outpatient rehabilitation programs. A key aspect of usual care is patient education and support from a multidisciplinary team that is directed at promoting self-care maintenance and monitoring behaviours. Healthy control participants will be living independently in the community without a medical diagnosis of HF and have adequate performance on the Addenbrooke’s Cognitive Examination Revised (ACE-R) [35].

### Participant exclusion criteria

Patients will be excluded if they: do not have access to a PC computer; do not have basic computer skills or do not have the physical ability to use a computer keyboard; reside in a long-term high-care facility (nursing home); have a documented history of moderate-to-severe cognitive impairment or dementia, a cerebrovascular accident, acute myocardial infarction or have undergone cardiac surgery in the previous 3 months; or have a terminal diagnosis. Carers will be excluded if they have a documented history of moderate-to-severe cognitive impairment or dementia or have a terminal diagnosis. Healthy control participants will be excluded if they: reside in a long-term high-care facility (nursing home); have moderate-to-severe cognitive impairment or dementia; or have experienced a cardiac event within the past 6 months (cerebrovascular accident, acute myocardial infarction or cardiac surgery).

### Recruitment

Participants will be recruited over a 24-month period, or until the required sample size is achieved. A HF nurse will initially assess patients’ eligibility to participate in the study: those who meet the inclusion criteria will be referred to the study research assistant who will approach potential participants at their outpatient appointment, explain the study (both verbal and written information), and obtain their consent. They will be asked to identify if they have an informal carer or next-of-kin who would be willing and able to complete some questionnaires for the study. A research assistant will contact the carer, explain the study and their involvement, obtain consent and collect baseline questionnaires. Healthy control participants will be sourced from the community by advertisements in local newspapers and word-of-mouth. Research assistants will screen control participants for study eligibility, explain the study and obtain their consent to undergo the neurocognitive testing. Healthy control participants will be matched to HF patients according to age, premorbid intelligence and gender.

### Baseline and follow-up measures to be collected from all HF patient participants

A comprehensive baseline assessment of HF patients will be conducted prior to randomisation and the first intervention session. The baseline assessment will include socio-demographic, psychosocial, cognitive and clinical profiling using a combination of self-report and review of patient records. Table 1 displays baseline, 4-week and 12-month assessments. The following measures will be administered during the assessment:

**Table 1:** **Assessment measures at baseline and post-intervention evaluations**

**Measures**	**Baseline**		
		**Assessment One** ^**b**^	**Assessment Two** ^**c**^
Physical functioning			
Physical examination (BP/HR/RR/weight/NYHA class, BNP)	X	X	X
Echocardiogram	X		X
‘Up and Go’ test	X	X	X
Timed Instrumental Activities of Daily Living (TIADL)	X	X	X
Unplanned readmission/survival		X	X
Self-care			
Heart-FaST	X		X
Patient engagement in self-care: (SCHFI and four consulting items from the EHFScBS)	X	X	X
Nurses and carer assessment of patient engagement in self-care	X	X	X
Review of patient symptom monitoring diary and how they perform self-care maintenance behaviours		X	X
Control Attitudes Scale - Revised (CAS-R)	X	X	X
Psychosocial functioning			
Quality of life (SF-12)^a^	X	X	X
Dyadic HF care typology	X		
Hospital Anxiety and Depression Scale (HADS)^a^	X	X	X
Cognitive functioning for HF patients			
Cognitive screen (MoCA)^a^	X	X	X
Assessment of PM (MIST, brief version of Virtual Week and naturalistic task)^a^	X	X	X
Working memory, executive functioning, verbal memory (CogState, N-Back, Trail Making Test)^a^	X	X	X
Premorbid intelligence (NART)^a^	X		
Caregiver			
Health screening questionnaire	X		
Caregiver (or next of kin) Contribution to Self-Care (CC-SCHFI)	X	X	X
Control Attitudes Scale - Revised (CAS-R)	X	X	X
Dyadic HF care typology	X		
Quality of life (SF-12)^a^	X	X	X
Modified Caregiver Strain Index (MCSI)	X	X	X
Proxy - IADL	X	X	X

Key: BNP, brain natriuretic peptide; BP, blood pressure; CC-SCHFI, Caregiver Contribution Self-Care Heart Failure Index; EHFScBS, European Heart Failure Self-care Behaviour Scale; Heart-FaST, Heart Failure Self-care Screening Tool; HF, heart failure; HR, heart rate; IADL, Instrumental Activities of Daily Living; MIST, Memory for Intentions Screening Test; MoCA, Montreal Cognitive Assessment; NYHA class, NART; National Adult Reading TestNew York Heart Association Classification; PM, prospective memory; RR, respiratory rate; SCHFI, Self-Care Heart Failure Index; SF-12, Short-Form 12-item health survey.

^a^Measures will also be collected from healthy non-HF controls at baseline; ^b^2 to 4 weeks post cognitive training; ^c^12 months post cognitive training.

I. *Patient clinical and socio-demographics*: age, gender, education level, social support, employment history. Patient clinical history, blood pathology (B-type natriuretic peptide (BNP), urea, electrolytes, full blood count), echocardiographic assessment of left ventricular systolic and diastolic function and assessment of valvular function, HF aetiology and treatments (medications, devices, surgery), physical examination (heart rate, blood pressure, respiratory rate, weight).
II. *Heart Failure Screening Tool* (Heart-FaST) [36]: assesses three functional domains salient to patient engagement in self-care: physical, cognitive and emotional functioning. Higher scores on each barrier scale indicate worse functioning. Preliminary analyses support the construct validity of the Heart-FaST as a screening tool for physical, cognitive and emotional barriers that may hinder engagement in HF self-care. Heart-FaST, therefore, has the potential to assist clinicians in tailoring educational and surveillance disease management strategies to individual need [37].
III. *Self-Care Heart-Failure Index* (SCHFI) [38]: assesses HF self-care behaviours and skills comprising 15 items rated on a 4-point scale to measure the process of self-care. There are three sub-scales: maintenance, management and confidence. Responses from each of the 3 self-care scales are transformed to a 100 point scale; higher scores reflect superior self-care. Self-care management scores are only computed for those patients reporting HF symptoms of ankle swelling or trouble breathing in the previous three months. Scaled scores > 70 are considered adequate self-care [38].
IV. *European Heart Failure Self-care Behaviour Scale* (*EHFScBS*) [39,40]: 4 items from this scale will assess consulting behaviours in response to increased dyspnoea, feet swelling, weight gain and fatigue. Scores on the consulting subscale range from 4 to 20 with higher values indicating a lower likelihood of contacting providers for guidance when a change in signs or symptoms suggests worsening HF.
V. *The Control Attitudes Scale - Revised* (CAS-R) [41]: assesses cardiac patients’ subjective perceived control in relation to managing their heart condition. Each of the 8 items is measured on a 5-point Likert scale ranging from 1 (strongly disagree) to 5 (strongly agree). Internal consistency in the HF population was Cronbach’s α = 0.76, and it showed good construct validity [41]. This scale will be administered to the HF patients, and will be adapted for administration to the caregivers. For example, one item on the CAS-R states: ‘If I do all the right things, I can successfully manage my heart condition’, and will be adapted for caregivers to state: ‘If I do all the right things, I can successfully manage the patient’s heart condition’. Similar amendments have been made in other studies investigating the perceived control of caregivers of patients with cardiac disorders [42,43].
VI. *Short-Form 12-item health survey* (SF-12) [44]: assesses quality of life. The health survey consists of 12 items on a Likert response: 1) physical functioning, 2) physical role, 3) bodily pain, 4) general health, 5) vitality, 6) social functioning, 7) emotional role, and 8) mental health. Summary scale scores can be transformed to a scale of 0 to 10, with higher scores representing better health [44].
VII. *The ‘Up and Go’ test* [45]: assesses functional mobility; stand from sitting, walk at usual speed for 3 meters, turn and return to sitting. To allow for test effect, an average of two timed trials will be recorded. The test is a measure of balance, walking ability and risk of falling in older populations and has been shown to be consistently reliable in test-retest analysis in patients with chronic conditions, including HF [45].
VIII. *Dyadic HF care typology* [46,47]: describes the caregiving relationship between the HF patient and nominated carer. Both the HF participant and their carer will, independently, identify how their relationship functions in terms of managing HF symptoms. Care relationship types have been operationalised into: 1) caregiver responsible for the majority of the care of the HF patient, 2) HF patient responsible for the majority of their own care, 3) caregiver and the HF patient equally responsible for all aspects of the care, or 4) caregiver and the HF patient responsible for different aspects of the care.
IX. *Hospital Anxiety and Depression Scale* (HADS) [48]: assesses anxiety and depression. Seven of the 14 items relate to anxiety and 7 relate to depression. Responses are provided on a Likert scale, and scores on each scale are interpreted in ranges: normal (0 to 7), mild (8 to 10), moderate (11 to 14), and severe (15 to 21). The 2 subscales have a mean correlation of 0.56, the mean Cronbach’s α is 0.83 for anxiety and 0.82 for depression [49].
X. *Montreal Cognitive Assessment* (MoCA) [50]: screens for mild cognitive impairment in the domains of: visual-spatial skills; executive functions; language; attention; concentration; working memory; memory recall; and orientation. Low educational attainment is corrected by adding 1 point to the participant’s final score for ≤ 12 years of formal education. The highest possible score is 30 and a score 26 and above indicates normal cognitive function [50].

In a second interview, HF patients will undergo comprehensive PM and neurocognitive assessment, and TIADL, which will take 90 to 120 minutes to complete. The second interview will include the following:

I. *Global cognitive screen*. The Addenbrooke’s Cognitive Examination Revised (ACE-R), a sensitive cognitive screening test of global cognitive functioning [35], measures 5 cognitive domains; attention/orientation, memory, verbal fluency, language and visuospatial abilities. Lower scores suggest poor cognitive performance. ACE-R is sensitive to early stages of dementia [51] and will be used as a participant screen. Adequate performance on the ACE-R is required for the participant’s data to be included in the final data sample.
II. *Prospective memory* (*PM*). There will be a rigorous assessment of PM.
  - The *Memory for Intentions Screening Test* (*MIST*) [52] is a standardised measure of PM developed for use in clinical settings. Throughout the test, participants will be required to remember and perform 4 time-based (for example; ‘in exactly 15 minutes please tell me it is time to take a break’) and 4 event-based tasks (for example: ‘when I hand you a red pen, please sign your name on the paper’). A series of word search puzzles will be provided as a distractor task to prevent overt rehearsal of the prescribed intentions. The researcher will monitor whether the participant remembers to perform the PM tasks correctly. Errors and scoring will be assessed in accordance with the MIST professional manual, which allows for an assessment of eight differing aspects of PM functioning. The MIST takes 30 minutes to administer. Total scores range from 0 to 48 with higher scores indicating better performance on the task. The MIST is sensitive to various neurological disorders, such as mild cognitive impairment [53] and has good reliability and validity [54].
  - The brief *Virtual Week assessment*, which involves 2 ‘virtual’ days of the computerised board game, will assess event-based and time-based PM tasks. Based on our previous study with HF patients [55], the brief version of Virtual Week will take 30 to 45 minutes to complete.
  - The *Naturalistic PM task* assesses 2 types of time-based PM tasks: 1) an appointment task at a set time of day and 2) one requiring monitoring a short interval of time. The latter is typical of many laboratory-based PM tasks. Participants will be asked to ring a prescribed number at 2 set times over 3 consecutive days. With each scheduled phone call, a recorded message will ask participants to call back after an interval of 5, 10 or 20 minutes.
III. *Timed Instrumental Activities of Daily Living* (*TIADL*). Participants’ level of independence in daily activities will be assessed using a modified version of the TIADL [56]. Participants are required to complete nine activities that are relevant to tasks they would perform at home. The original five tasks are functional measures basic to independent living such as telephone communications, financial abilities, nutrition, shopping and medication use. Four additional tasks that are more relevant in the current technological communication environment and specifically related to HF self-care have been adapted for this study: 1) checking and sorting the sodium content of 3 food packages from the lowest to the highest amount of sodium; 2) extracting health information from a computer screen simulating a webpage to estimate a target heart rate for exercise; 3) extracting data from a webpage to look up a specified train timetable, and 4) finding a matching pair of black socks in a laundry basket full of mixed single socks. Participants are scored on the length of time to complete each of the nine activities, and if any, the type of error in performing the activity. Shorter completion times indicate better performance. The rapid and efficient completion of these tasks is advantageous in daily life and the time taken to complete the tasks is an objective indication of functional independence [56]. This assessment will be a key strength to the study, providing an objective and sensitive behavioural measure to evaluate the effectiveness of the cognitive training intervention on everyday functional independence.
IV. *Working memory and executive functioning*. A battery of tests from the CogState program will be used (www.cogstate.com) [57,58]: i) The One Back Task assesses working memory. Participants are presented with a succession of playing cards on a screen and must decide if the card displayed is the same as the one presented immediately before; ii) The Two Back Task is presented in the same way as the One Back Task: however, participants are required to decide if the card displayed on screen is the same as a card presented two cards previously. Responses that are more accurate reflect better working memory, on both tasks; iii) The Detection Task measures psychomotor functioning and speed of processing. Participants must respond as quickly as possible to a card flipping over on screen, by pressing a button on the keyboard. Reaction time is measured and lower scores indicate better performance; iv) The Identification Task measures visual attention. Participants must decide whether a playing card presented on screen is red, by pressing the ‘Yes’ or ‘No’ button. Reaction time is measured and lower scores indicate better performance; v) The One Card Learning Task measures visual learning and memory. Participants are presented with a succession of playing card on screen, and must decide if the card currently displayed has been displayed previously. Accuracy of performance is measured, with higher scores indicating better performance. In addition to the computer battery of tests that take only 8 minutes to complete, there will be a pen and paper task, the Trail Making Test (TMT) [59], which assesses planning ability. It consists of two parts: Part A and B. In Part A, participants are required to draw lines to connect circles that are numbered consecutively; in Part B, participants connect circles that are numbered or lettered, alternating between the numeric and alphabetic sequences. This test takes approximately 5 minutes to complete, and the participant’s score is the total time taken to complete the task. Lower scores on the TMT indicate higher levels of planning ability.
V. *Verbal memory*. In the Hopkins Verbal Learning Test-Revised [60,61], participants must listen to a list of 12 words read out by the experimenter, and then verbally recall as many words as possible from that list. This same procedure is repeated two more times. The participant is then asked, after a 20- to 25-minute interval, to verbally recall the same list of words. Following this final trial, the researcher reads out a list of 24 words and the participant is asked to identify the words that were presented in the original list of 12 words. This verbal memory test assesses episodic memory and the delayed recall test is particularly sensitive to ageing and abnormal ageing. The test has been successfully used in clinical and healthy populations of older adults [62].
VI. *Premorbid intelligence*. The National Adult Reading Test (NART) [63] is a word-recognition test of vocabulary knowledge that requires participants to read aloud 50 English words of increasing difficulty that do not follow normal phonetic rules: for example, ‘chord’. This test takes about 5 minutes to complete and responses are audio-recorded for scoring purposes. The NART is widely used as an estimate of premorbid ability given the premise that reading ability is relatively independent of brain damage. Furthermore, NART performance appears to be impervious to the effects of many neurological and psychiatric conditions [64]. In a retrospective validity study of the NART, there was a strong correlation (*r* = 0.73; *P* < 0.001) between Intelligence Quotient (IQ) age 11 and NART performance at age 77 [64]. The NART will be used to characterise the HF sample and match the healthy non-HF controls on premorbid intelligence.

### Baseline and follow-up measures to be collected from carers

Carers will be asked to complete seven brief questionnaires:

I. A health screening questionnaire will be used to identify the presence of specific health problems (cardiac, metabolic, diabetic, respiratory, muscular, family history, treatment precautions, and physical activity) and will be used for descriptive purposes.
II. Carer contribution to HF self-care (CC-SCHFI) [65] is derived from the SCHFI and assesses the career’s contribution to self-care maintenance and management behaviours. This information will be used to provide a more comprehensive assessment of the patient participant’s engagement in self-care.
III. Control Attitudes Scale - Revised (CAS-R) [41] will be administered to measure the carer’s perceived control in relation HF management. Each item is measured on a 5-point Likert scale ranging from 1 (strongly disagree) to 5 (strongly agree) and has been shown to have good internal reliability in a HF population (α = 0.76) [41]. This scale has been adapted for administration to the caregivers of patients with cardiac disorders [42,43].
IV. Carers will also identify how their relationship with the HF patient functions in terms of managing HF symptoms using the dyadic HF care typology (described above).
V. SF-12 will be administered (described above) as an indication of the quality of life of the carer.
VI. Modified Caregiver Strain Index (MCSI) assesses the causes, and the degree of strain, and changes in strain over time. It has four domains of strain: financial, physical, psychological and social and personal. The reported internal reliability of the MCSI is high (Cronbach’s α = 0.90) and it correlates with care recipient’s functioning [66,67]. The tool will be administered to monitor changes in carer strain from baseline to 3- and 12-month follow-up.
VII. To measure of HF patients’ level of functioning, carers will be asked to indicate the HF participants’ level of independence in daily activities using the validated Instrumental Activities of Daily Living (IADL) Scale [68]. This instrument assesses how much help the individual requires to perform each of eight tasks (using the telephone, shopping, food preparation, housekeeping, laundry, transportation, managing medications and home finances). A score of 1 indicates ‘no help’, and higher scores indicate increased dependence. Performing instrumental daily activities successfully requires a high level of skill and judgment: therefore, greater dependency is likely to be sensitive to mild changes in cognitive capacity [69].

### Measures to be collected from healthy control group

The healthy control group will be matched to the HF patient group on age, premorbid intelligence and gender, and will undergo neuropsychological assessment as described above and highlighted with asterisks in Table 1. Psychosocial demographics, including social support and anxiety and depression, will also be collected to allow comparisons of the healthy controls and HF participants.

### Additional patient outcome measures

There will be 3 additional outcome measures of self-care collected at Assessments One (2 to 4 weeks post cognitive training) and Two (12 months post cognitive training):

The first is patient engagement in self-care. On enrolment into the study, HF patients will be provided with a symptom monitoring diary by the research assistant who will explain how to use them. At Assessments One and Two the research assistant will review whether the diaries have been completed. The research assistant will also collect practical data on how the HF patient performs daily weighing, monitors their fluid intake; reads food labels to determine the sodium content of food; and follows their prescribed medication regimen.

The research assistant will ask the HF nurse caring for the patient participant to complete a questionnaire to assess the nurses’ perception of the patient’s engagement in HF self-care.

The *Unplanned Healthcare Utilisation Questionaire* will be used to collect information about unscheduled visits to GP, cardiologist, HF nurse and emergency department. Information about survival will be collected from hospital records.

### Outcomes

Primary study outcomes are patient engagement in HF self-care behaviours from both self-report (SCHFI) and the objective assessments from the carer, researcher and nurse, described above. Secondary outcomes are those not directly related to HF self-care behaviors but relevant to HF: quality of life; BNP, ‘Up and Go’ test, hospital readmissions, survival and transfer effects. To examine near to far transfer effects of the cognitive training we shall use PM tests aligned (or similar) to the training task, MIST and Virtual Week for near transfer effect. To examine for far transfer effects we will reassess overall cognitive function at Assessments One and Two. The TIADL and naturalistic PM task will assess transfer to everyday functioning, and will potentially represent somewhere between near and far transfer.

### Interventions

Included in this study are 2 interventions: 1) PM training intervention (Virtual Week Training Program) and 2) the active control intervention (non-adaptively difficult word puzzles). The active control intervention is active in the sense that participants undergo an intervention that they may perceive as a plausible technique for improving cognition, but which is not evidenced to do so, similar to a placebo control. Relative to a passive control, such as usual care in a medical context, an active control is a more rigorous test of the effects of cognitive training.

### PM training intervention - Virtual Week Training Program

HF patients randomly allocated to receive the computer-based PM training strategy (Virtual Week Training Program) will continue to receive their usual care from service providers; this may be from any combination of nurse-led chronic disease management, specialised HF outpatient clinics, or outpatient rehabilitation programs. As a supplement to usual care, the Virtual Week Training Program intervention will have all the critical features of a restorative training task with lots of repetition and adaptive difficulty whereby the task starts at a relatively easy level and then progressively becomes more difficult, adapting to the performance level achieved by the participant. A key feature of this program is the distributed practice of the cognitive training, in this case over 6 weeks, rather than massed practice in 1 or 2 long sessions. Another training feature is that the Virtual Week Training Program provides feedback at the conclusion of each ‘virtual day’ (which will occur twice per session), so that participants are aware of their performance on each day and of their progression through the difficulty levels. Previous training tasks have mixed success with often low levels of far transfer, and this is arguably because the training tasks have not been specifically targeted towards desirable outcome behaviours. We have developed ‘virtual days’ that mimic many of the daily tasks HF patients would be expected to follow in real life. For example, when patients participate in the Virtual Week Training Program, they are asked to simulate activities like weighing themselves daily in the morning, evaluating their HF symptoms in the evening, and assessing their fluid intake at several times during the day. In addition, there are event-based tasks that involve exercise. Therefore, the Virtual Week Training Program is more related to PM than many previous restorative training programs that train performance on basic cognitive tasks, such as working memory [33].

The Virtual Week Training Program has been substantially developed since the reported pilot [33]. The number of ‘virtual days’ has been doubled and each of the 48 days contains a different set of event cards to simulate unique sets of daily activities and PM tasks. This avoids the repetition of ‘virtual days’ that was needed in the pilot and extends the sessions from 12 sessions over 4 weeks to 24 sessions over 6 weeks. The training program is now fully automated so that participants can complete the training sessions in their own homes without needing to travel to the laboratory for supervised administration.

The research assistant conducting the training sessions will be blinded to baseline assessments. It is anticipated that the first training session will take 40 to 45 minutes where the patient will be provided with a USB version of the Virtual Week Training Program to use on their home computer over 6 weeks. Patients will be instructed to use the training program 4 times per week over 6 weeks. Each training session will take approximately 20 to 30 minutes to complete. On insertion of the flash drive into the patient’s computer, prompts will initiate the training at the appropriate level and limit the number of ‘virtual days’ (2) that can be completed in 1 session and the number of sessions that can be completed on each actual day (1) and each actual week (4). The Virtual Week Training Program records all responses, scores multiple measures of performance, and uploads the data to the experimenter’s database at the completion of each session so that the patient’s progress can be monitored. Face-to-face support will be arranged where needed.

### Active control group - puzzle training

To determine whether cognitive training has any additional health benefits for HF patients there will be an active control group to enable comparisons of outcome measures between the two HF groups. HF patients randomly allocated to the active control will continue to receive their usual care from service providers; this may be from any combination of nurse-led chronic disease management, specialised HF outpatient clinics, or outpatient rehabilitation programs. This condition will involve participants completing computerised word puzzles, and will be presented not as a control activity but as a plausible training activity. This particular control has been implemented due to the widespread belief that ‘brain teasers’ (for example, crossword puzzles) help with brain fitness, yet evidence for the efficacy of commercial brain-training exercises is limited [70].

To control for the social and participatory features of the PM training, patient participants in the control group will be asked to complete a set of word puzzles for a similar amount of time to the restorative memory training condition. That is, they will be asked to spend 20 to 30 minutes per day completing a set of word puzzles, 4 times per week, over 6 weeks. The key distinguishing features of the word puzzles compared to the Virtual Week Training Program is that the puzzle difficulty is manageable and non-adaptive. Compared to the PM training group, patient participants in the control group will have similar levels of contact with the research assistant: the introductory sessions will be conducted face-to-face to introduce them to the puzzle training; subsequent telephone contact twice a week to support and monitor their participation in the puzzle training. Patient participants will be given specific instructions and support to Email screenshots to the research assistant to monitor their participation and progress.

### Randomisation and allocation concealment

**Figure 1:**
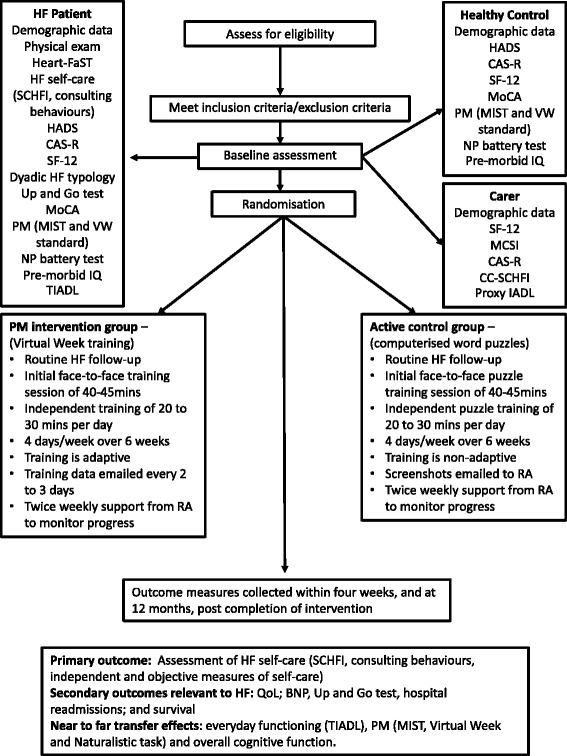
Consolidated Standards of Reporting Trials (CONSORT) diagram illustrating study procedures. Key: BNP, brain natriuretic peptide; CAS-R, Control Attitudes Scale - Revised; CC-SCHFI, Caregiver Contribution Self-Care Heart Failure Index; CRA, Caregiver Reaction Assessment; Dyadic HF Typology, Dyadic Heart Failure Typology; HADS, Hospital Anxiety and Depression Scale; Heart-FaST, Heart Failure Self-care Screening Tool; HF, heart failure; MIST, Memory for Intentions Screening Test; MCSI, Modified Caregiver Strain Index; MoCA, Montreal Cognitive Assessment; NP-battery test, Neuropsychological battery test; Proxy IADL, Proxy measure Instrumental Activities of Daily Living; SCHFI, Self-Care Heart Failure Index; TIADL, Timed Activities of Daily Living.

Using CONSORT principles [71] HF patients will be randomised to 1 of the 2 study interventions: 1) usual care plus PM training (see Figure 1) or 2) active control group. Block randomisation will be allocated via a computer generated number prepared by an investigator with no involvement in data collection. Block randomisation with 1:1 ratio will be used to keep the 2 groups at similar sizes. Once the HF patient has consented to the study and baseline measures have been collected the research assistant will contact one of the training study investigators, who are independent of the recruitment process and data collection, for allocation consignment. Research assistants collecting baseline measures will not be informed of the patient’s assignment. Instead, the research assistant conducting the cognitive training will be notified when a patient has been randomised to this intervention. Participants enrolled in the study will be aware of the training activity they have been allocated and therefore they will not be blinded to the treatment allocation.

The study has received ethical approval from the St Vincent’s Hospital, Melbourne (HREC-A 018/14) and Australian Catholic University (Ethics Register Number: 2014 175 V) Human Research Ethics Committees and will conform to the principles outlined in the *Declaration of Helsinki* and to the Consolidated Standards of Reporting Trials (CONSORT) guidelines for a randomised controlled study comparing the efficacy of two non-pharmacological health interventions [72]. The trial is registered with the Australian New Zealand Clinical Trials Registry (ANCTR: reference number #366376). All subjects will provide written informed consent to participate in the study.

### Sample size

Based on data from other relevant trials of patient education and HF self-care, an eight-point difference in standardised self-care scores is a clinically important change [73]. Using the G*power calculator it was estimated that in order to achieve an 80% power at a 0.05 significance, a sample size of 63 per group is required to detect a small to medium effect of the self-care intervention on hospitalisations [73]. Using analysis of covariance (ANCOVA) to examine for clinically significant differences in standardised self-care maintenance and management scores between the 2 HF groups, a total of 100 patients in each group (200 in total) will have > 90% power (α of 0.05) to detect a medium effect size (f^2^ = 0.4) in the primary endpoint. The probable requirement to undertake non-parametric statistical analyses (due to non-Gaussian distributed data) has been factored into study power calculations in addition to a 20% intervention refusal rate and loss to follow-up. The sample size for the comparison of baseline cognitive performance (in particular PM) between 60 HF patients and a healthy age-matched control group has been based on previous studies [55,74]. As such, the sample size has the power to detect significant differences with moderate effect sizes.

### Statistical analyses

To compare and confirm cognitive deficits, including PM, between HF patients and a healthy matched control, group differences in baseline PM and neuropsychological tests will be examined using inferential statistics (*t*-test and analysis of variance; ANOVA). To analyse the changes in each measure assessed at each time point (baseline, Assessment One and Assessment Two) we will use separate mixed ANOVAs for all dependent variables. The within-groups variable will be *testing phase* (baseline, Assessment One, Assessment Two) and the between-groups variable will be *Training group* (PM training, word puzzles). Multiple regression analysis will be performed to identify whether physical, cognitive, emotional and mental functioning variables are independent predictors of HF self-care and consulting behaviours, after adjusting for clinical characteristics. For all analyses, α levels will be set at 0.05 and effects sizes will refer to partial η-square values.

### Dissemination

The dissemination plan includes five scientific papers that will be submitted to high-quality, peer-reviewed medical, nursing and psychology journals:

1. *Prospective memory, a missing link in explaining effective engagement in HF self-care*
2. *Changes in HF self-care behaviours following a 6-week cognitive training program using the Virtual Week Training Program: a randomised controlled study*
3. *Can a 6-week, computer-based cognitive training program improve quality of life and reduce hospital admissions in HF? A randomised controlled study*
4. *Does prospective memory change in HF patients following a 6-week cognitive training program using the Virtual Week Training Program? A randomised controlled study*
5. *Do markers of HF severity change with improved engagement in self-care behaviours? A randomised controlled study*.

## Discussion

Cognitive impairment is a significant problem that adds to the burden of living with HF. With a growing interest in PM in ageing [14,75-77] and various other clinical conditions [15,78-81], this study may further build on this knowledge, increasing not only our understanding of cognitive impairment in HF but also possible management strategies. Although there is encouraging evidence for the impact of cognitive training in ageing [33,82-88], few studies have investigated cognitive training in HF as a possible solution to improve health outcomes [89]. This study will be the first to explore if PM is associated with HF self-care and whether the Virtual Week Training Program, a novel computer-based restorative memory training program, improves functional outcomes relative to receiving usual care alone. The strength of this study will be rigorous baseline measurement of cognitive function and self-care, and expected improvements through novel and comprehensive insights into the prediction of HF self-care and trajectories when education, support and cognitive training are applied on an individually tailored basis.

Results from this study may lead to an innovative treatment by means of cognitive training, to optimise HF patients’ engagement in self-care abilities, such as improving adherence to medications, diet and exercise. Participation in this study will not result in any known adverse events: however, potential benefits to patients include improved wellbeing, and enhanced skills in managing HF symptoms and as such may impact on hospital readmissions. In this proposed study, patients will be randomly allocated to receive either the Virtual Week Training Program or the non-adaptive computer-based word puzzles. Both groups will be expected to complete their allocated training program on 4 days of the week for 6 weeks. A novel feature of the study is the collection of not only self-reported self-care but some objective measures from carers and HF nurses, and objective measures of functional independence such as TIADL. Self-reports rely heavily on memory demands which, if diminished, suggest that questionnaires may not be accurate. Furthermore, the comprehensive assessment of patient functioning (BNP, ‘Up and Go’ test, activities of daily living) will enable us to investigate if small improvements in PM have an incremental impact on physical functioning and quality of life. In conclusion, we anticipate that cognitive training will improve health outcomes in HF patients and reduce associated healthcare costs.

## Trial status

This study is actively recruiting and ongoing.

## Abbreviations

ACE-R: Addenbrooke’s Cognitive Examination Revised
ANCOVA: analysis of covariance
ANOVA: analysis of variance
BNP: brain natriuretic peptide
CAS-R: Control Attitudes Scale - Revised
CC-SCHFI: Caregiver Contribution Self-Care Heart Failure Index
CONSORT: Consolidated Standards of Reporting Trials
CRA: Caregiver Reaction Assessment
EHFScBS: European Heart Failure Self-care Behaviour Scale
HADS: Hospital Anxiety and Depression Scale
Heart-FaST: Heart Failure Self-care Screening Tool
HF: heart failure
IQ: Intelligence Quotient
MCSI: Modified Caregiver Strain Index
MIST: Memory for Intentions Screening Test
MoCA: Montreal Cognitive Assessment
NART: National Adult Reading Test
NP-battery test: Neuropsychological battery test
PM: prospective memory
Proxy IADL: Proxy measure Instrumental Activities of Daily Living
SCHFI: Self-Care Heart Failure Index
SF-12: Short-Form 12-item health survey
TIADL: Timed Activities of Daily Living
TMT: Trail Making Test
